# Effects of enamel matrix derivative on the proliferation and osteogenic differentiation of human gingival mesenchymal stem cells

**DOI:** 10.1186/scrt441

**Published:** 2014-04-16

**Authors:** Shu-Man Wu, Hsien-Chung Chiu, Yu-Tang Chin, Heng-Yi Lin, Cheng-Yang Chiang, Hsiao-Pei Tu, Martin MJ Fu, Earl Fu

**Affiliations:** 1 Department of Periodontology, School of Dentistry, National Defense Medical Center and Tri-Service General Hospital, PO Box 90048–507, Taipei, Taiwan; 2 Department of Dental Laboratory Technology, Min-Hwei College of Health Care Management, Tainan County 736, Taiwan; 3 Taipei Cancer Center, Taipei Medical University, Taipei, Taiwan; 4 Dental Department, Cardinal Tien Hospital, New Taipei City, Taiwan; 5 Department of Dental Hygiene, China Medical University, Taichung, Taiwan; 6 Division of Periodontology, Department of Oral Medicine, Infection and Immunity, Harvard School of Dental Medicine, Boston, MA, USA; 7 School of Dentistry, National Defense Medical Center, PO Box 90048–507, Taipei, Taiwan

## Abstract

**Introduction:**

Gingiva-derived mesenchymal stem cells (GMSCs) have recently been harvested and applied for rebuilding lost periodontal tissue. Enamel matrix derivative (EMD) has been used for periodontal regeneration and the formation of new cementum with inserting collagen fibers; however, alveolar bone formation is minimal. Recently, EMD has been shown to enhance the proliferation and mineralization of human bone marrow mesenchymal stem cells. Because the gingival flap is the major component to cover the surgical wound, the effects of EMD on the proliferation and mineralization of GMSCs were evaluated in the present study.

**Methods:**

After single cell suspension, the GMSCs were isolated from the connective tissues of human gingiva. The colony forming unit assay of the isolated GMSCs was measured. The expression of stem cell markers was examined by flow cytometry. The cellular telomerase activity was identified by polymerase chain reaction (PCR). The osteogenic, adipogenic and neural differentiations of the GMSCs were further examined. The cell proliferation was determined by MTS assay, while the expression of mRNA and protein for mineralization (including core binding factor alpha, cbfα-1; alkaline phosphatase, ALP; and osteocalcin, OC; ameloblastin, AMBN) were analyzed by real time-PCR, enzyme activity and confocal laser scanning microscopy.

**Results:**

The cell colonies could be easily identified and the colony forming rates and the telomerase activities increased after passaging. The GMSCs expressed high levels of surface markers for CD73, CD90, and CD105, but showed low expression of STRO-1. Osteogenic, adipogenic and neural differentiations were successfully induced. The proliferation of GMSCs was increased after EMD treatment. ALP mRNA was significantly augmented by treating with EMD for 3 hours, whereas AMBN mRNA was significantly increased at 6 hours after EMD treatment. The gene expression of OC was enhanced at the dose of 100 μg/ml EMD at day 3. Increased protein expression for cbfα-1 at day 3, for ALP at day 5 and 7, and for OC at week 4 after the EMD treatments were observed.

**Conclusions:**

Human GMSCs could be successfully isolated and identified. EMD treatments not only induced the proliferation of GMSCs but also enhanced their osteogenic differentiation after induction.

## Introduction

Mesenchymal stem cells (MSCs) are multipotent progenitor cells derived originally from adult bone marrow or some adult/fetal non-marrow tissues. Over recent years, several different MSCs have been harvested and identified from various dental tissues, including dental pulp stem cells [1,2], stem cells from exfoliated primary teeth [3], periodontal ligament stem cells [4], dental follicle precursor cells from wisdom teeth [5], stem cells from periapical follicle [6] and gingiva-derived mesenchymal stem cells (GMSCs) [7]. Because of their capabilities of multipotent differentiations, dental stem cells have been suggested as a potential candidate for tissue engineering and/or regenerative medicine. They can be used not only for regenerating dental tissues, but also for repairing non-dental tissues, such as bone and nerves [8,9].

Periodontal disease is a common bacteria-associated inflammatory disease. The infective and inflammatory reactions from periodontal disease may damage the surrounding hard and soft tissue structures, called the periodontal attachment which requires alveolar bone, periodontal ligament and cementum, and results in tooth loss in the end [10,11]. Numerous materials have been utilized to improve the regenerative treatment outcome of periodontal disease, including enamel matrix derivative EMD (Emdogain®, Institut Straumann AG, Basel, Switzerland). EMD (Emdogain®) is a purified acidic extract from the enamel matrix protein of the tooth bud and predominantly consists of amelogenins [12]. The application of EMD in periodontal regenerative treatment has been widely focused on its ability to promote the formation of the lost periodontal attachment, especially on the regeneration of the periodontal ligament and cementum [13,14]. Some studies have reported that EMD can also stimulate cellular proliferation and mineralization of pre-osteoblasts and osteoblasts [15-18]. On the other hand, some studies have reported that EMD reduces the differentiation of osteoblasts [19,20]. Although a significant amount of new cementum has been widely observed following EMD application during periodontal treatments, the alveolar bone formation is reported to be minimal [21,22].

To rebuild the lost periodontal attachment is the ultimate goal of periodontal therapy; however, true regeneration after the therapy is still challenging [9]. Recently, dental stem cells have been reported as candidates to restore the lost periodontal tissue [23]. In addition, it has recently been reported that EMD enhances the proliferation and mineralization of human bone marrow MSCs [24]. Clinically, the EMD is applied to the tooth surfaces during periodontal regenerative surgery and covered with a gingival flap which is the main source of GMSCs. The effects of EMD on stem cells, especially on those derived from the local gingiva, have never been evaluated. The aim of the present study was to investigate the effects of EMD on the proliferation and mineralization of GMSCs.

## Methods

### Human gingiva-derived mesenchymal stem cells (hGMSCs)

Gingival tissue specimens were obtained from patients treated in the Periodontal Department of Tri-Service General Hospital from July 2009 to December 2010. The specimens were taken from either crown lengthening procedures or distal wedge periodontal surgeries. After being stored in an alpha modification of Eagle’s medium (α-MEM) (Invitrogen, Grand Island, NY, USA) with 10% qualified fetal bovine serum (FBS) (Invitrogen) and 1% penicillin, streptomycin (P/S), the specimens were immediately transported to the laboratory. In the present experiments, all of the procedures had been approved by the Ethics Committee of the Faculty of Medicine, Tri-service General Hospital, Taipei, Taiwan (TSGHIRB-100-05-099) and all participants gave their informed consent.

**Figure 1:**
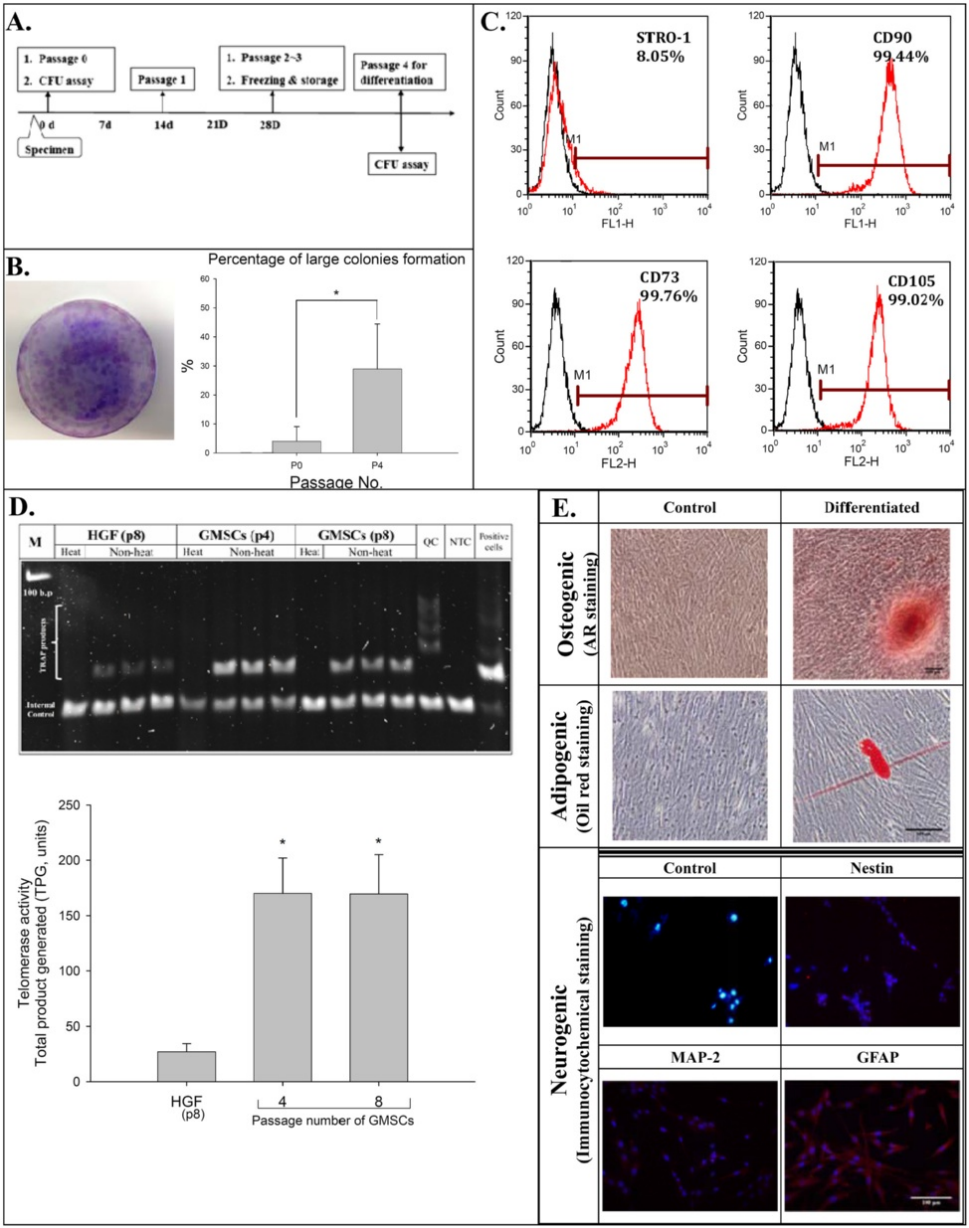
**The isolation and characterization of hGMSCs. (A)** The isolation protocol/timeline of hGMSCs is illustrated. **(B)** The colony of isolated hGMSCs was stained with crystal violet. **(C)** The mesenchymal stem cell surface markers, which are CD90, CD73 and CD105, were highly expressed in hGMSCs, but STRO-1 was expressed at a low level. **(D)** The telomerase activity of hGMSCs was significantly higher than that of gingival fibroblasts. **(E)** Multi-potency in the differentiation of these isolated hGMSCs was successfully induced and characterized, including the osteogenic, adipogenic and neural differentiations. hGMSCs, human gingiva-derived mesenchymal stem cells.

The method for stem cell isolation was modified from previously described procedures [4]. Briefly, the connective tissue was separated from the epithelium after overnight treatment with Dispase II (Roche Diagnostics, Indianapolis, IN, USA). Then, the connective tissue was digested with 0.2% collagenase (Sigma-Aldrich Inc., St. Louis, MO, USA), and the cell suspension of gingival fibroblasts was collected. After being filtered through a 70 μm strainer, the cells were cultured at 1 × 10^5^ cells/10 cm culture dish with the α-MEM at 37°C and 5% CO_2_ (Figure 1A). In this study, passage 0 (P0, primary culture) cells, the hGMSCs, were collected at this stage. The passage 1 (P1) cells were collected two weeks later and a series of subcultures was then performed after confluence. In order to determine the characteristics of the isolated cells, the colony forming efficiency (the self-renewal ability) and telomerase activity (the cell proliferation capability), as well as the surface expression of stem cells markers, were assayed.

### Colony forming unit assay

The efficiency of hGMSCs in forming colonies was detected with a colony forming unit (CFU) assay. P0 cells were plated at 5,000 cells/10 cm dish and P4 cells were plated at 500 cells/10 cm dish. After being cultured for 14 days, they were fixed and stained with crystal violet. The number and size of the colonies, containing 50 or more cells, were recorded. The colony forming rate was then calculated as the number of colonies formed per hundred cells.

### Telomerase activity assay

To examine the cell proliferation capability of hGMSCs, a telomerase activity assay was carried out. The telomerase activity of hGMSCs was measured with 1.5 μg of protein extracts using a TRAPEZE® Telomerase Detection Kit (S7700, Merck Millipore Headquarters, Billerica, MA, USA), and the protein concentration was determined with a BCA™ Protein Assay Kit (Thermo Fisher Scientific Inc., Waltham, MA, USA). TRAP assay products were separated on a 10% polyacrylamide gel following staining with SYBR® Safe staining (Invitrogen) and were visualized with a camera system (ChemiDoc XRS + system, Bio-Rad Laboratories, Hercules, CA, USA). The gel images were scanned directly with software (Image Lab, Bio-Rad Laboratories) and quantitated according to the kit instructions.

### Flow cytometry for surface marker analysis

To determine the expression of the conventional surface markers used to define hMSCs (CD73, CD90 and CD105) and stromal stem cells (STRO-1) on hGMSC, they were examined by flow cytometry. The hGMSCs were prepared as single cell suspensions by trypsinization and resuspended in blocking buffer containing Hank’s balanced salt solution (Sigma–Aldrich Inc.) supplemented with 1% bovine serum albumin (BSA; Sigma–Aldrich Inc.) for 30 minutes. Approximately 1 × 10^6^ cells/mL were incubated with phycoerythrin (PE)- or fluorescein isothiocyanate (FITC)-conjugated monoclonal antibodies against CD73 (BD Biosciences, San Jose, CA, USA), CD90 (eBioscience, San Diego, CA, USA), CD105 (eBioscience) and STRO-1 (Santa Cruz Biotechnology, Santa Cruz, CA, USA, ) for 30 minutes at 4°C in the dark, then rinsed and kept in Hank’s balanced salt solution with 1% BSA on ice until analysis. Samples were analyzed using a FACSCalibur flow cytometer (Beckman Coulter, Hialeah, FL, USA). Data were processed using FCS Express V3 software (Beckman Coulter).

### Osteogenic, adipogenic, neural differentiations of hGMSCs

Osteogenic differentiation was induced after the hGMSCs were seeded at a density of 1 × 10^4^ cells/well on 12-well culture plates in α-MEM with 5% FBS and 1% P/S. When cells reached 80% confluence, the medium was changed to osteogenic medium which contained α-MEM with 5% FBS, 1 nM dexamethasone (Sigma-Aldrich Inc.), 50 μM L-ascorbic acid 2-phosphate sesquimagnesium salt (Sigma-Aldrich Inc.), 20 mM β -glycerophosphate (Sigma-Aldrich Inc.) and 50 ng/mL L-thyroxine sodium pentahydrate (Sigma-Aldrich Inc.). The medium was changed twice a week for three weeks for osteogenic induction. After being fixed with 4% paraformaldehyde, the culture was stained with 1% Alizarin Red S at pH 4.1 for 20 minutes.

For adipogenic differentiation, 1 × 10^4^ cells/well were seeded on 12-well culture plates. When the cells reached 80% confluence, the medium was changed to adipogenic medium which contains α-MEM with 5% FBS, 1 μM dexamethasone, 50 μM indomethacin (Sigma-Aldrich Inc.), 5.0 μg/mL insulin (Sigma-Aldrich Inc.) and 0.5 μM 3-isobutyl-1-methylxanthine 3-isobutyl-1-methylxanthine (IBMX, Sigma-Aldrich Inc.). Then the medium was changed twice a week for four weeks for adipgenic induction. After fixation, the culture was stained with 0.0125% Oil red O (Sigma-Aldrich Inc.) in isopropanol for 20 minutes.

For neural differentiation, 1 × 10^4^ hGMSCs/well were seeded on poly-L-lysine/laminin-coated eight-well multiple-chamber slides (Merck Millipore) in Dulbecco^’^s modified Eagle^’^s medium/F12 (DMEM/F12) (InvitroGen) supplemented with 125 ng/ml basic fibroblast growth factor (bFGF) (InvitroGen), 1,000 unit/ml leukemia inhibitory factor (Sigma-Aldrich Inc.) and 4 mM forskolin (Sigma-Aldrich Inc.). After three to approximately seven days, the cells were fixed with 4% paraformaldehyde for immunocytochemistry staining.

### EMD treatment, the cell proliferation assay and osteogenic differentiation

To evaluate the effects of EMD on the proliferation of hGMSCs, 1 × 10^4^ cells were cultured in a well containing 10% FBS in α-MEM media (Invitrogen Corporation, Carlsbad, CA, USA) to 70% confluence in a 96-well plate, and the medium was changed to serum-free α-MEM (InvitroGen, Grand Island, NY, USA) to starve the cells overnight. The cells were then treated with 0, 25 or 100 μg/ml of EMD (Emdogain®; Straumann AG, Basel, Switzerland) for 24 hours or 48 hours. The proliferation of hGMSCs was measured at OD 490 nm using the CellTiter 96® AQueous One Solution Cell Proliferation Assay (MTS, Promega, Madison, WI, USA). All the experiments were repeated three times.

To examine the osteogenic effects of EMD on hGMSCs, the stem cells were cultured at a density of 1 × 10^4^ cells/well in 12 well culture plates and 1 × 10^3^ cells/well in eight-well chamber slides (Merck Millipore) in α-MEM with 5% FBS and 1% P/S (Invitrogen) until 70% confluence. The cells were treated with a mineralization solution (50 μM ascorbic acid, 10 nM dexamethasone and 20 mM β-glycerophosphate) (Sigma-Aldrich) either in the absence of EMD (control cultures) or in the presence of EMD (25 μg/ml or 100 μg/ml). Then, the medium was changed twice a week. After one, two, three or four weeks of cultivation in each treatment, hGMSCs were washed with PBS and fixed with 4% paraformaldehyde (Sigma-Aldrich). The cultured cells in the 12-well culture plate were stained with Alizarin red S (ARS) (Sigma-Aldrich) following routine procedures. Later, 0.5 N HCl and 5% sodium dodecyl sulfate were added to each well to dissolve the stained nodules. The light absorbance of the extracted dye was measured with a microplate spectrophotometer (Thermo Fisher Scientific) at 405 nm as previously described [25]. The cultured cells in the eight-well chamber slides were used for immunocytochemistry staining.

### Reverse transcription polymerase chain reaction and real-time PCR

To explore the expressions of osteogenic genes, hGMSCs were seeded at a density of 1 × 10^5^ cells/well in six-well culture plates, and the effects of different concentrations of EMD on gene expression were observed. The treatment conditions were identical to those of the osteogenic differentiation.

After various times (including 3, 6, 12, 24 or 72 hours) of cultivation and treatment with EMD or not, RT-PCR was performed to evaluate the semi-quantity of gene expression in alkaline phosphatase (ALP) and osteocalcin (bone γ-carboxyglutamate (Gla) protein; OC). To measure the mRNA expression of ALP and OC after osteogenic treatment with or without EMD, total RNA was extracted using TRIsure reagent (Bioline Ltd., London, UK). As described previously [26] with slight modification, 1 μg of total RNA was reverse transcribed with Tetro RT enzyme (Bioline Ltd.) into cDNA, and used as the template for PCR reactions and analysis.

Transcribed cDNA was then amplified using the QuantiTect Primer Assay gene expression assay, including QT00020517 for Cbfα-1, QT00012957 for ALP, QT00232771 for OC and QT01192646 for the endogenous control glyceraldehyde-3-phosphate dehydrogenase (GAPDH) according to the manufacturer’s instructions using a Rotor-Gene cycler (QIAGEN, Hilden, Germany). Gene expression for human ameloblastin (AMBN), forward 5′-GAGTTTTGCAGTGCCGTTCT-3′ and reverse 5′-CTGCAGACTTCCCAACTGTCT-3′ (NM_016519.5) was also determined. Calculations of relative gene expressions (normalized to GAPDH reference gene) were performed according to the 2^-ΔΔCT^ method [27].

### Alkaline phosphatase enzyme activity assay

We also addressed osteogenic differentiation of hGMSCs by measuring ALP activity in culture. hGMSCs were cultured as described above, either without EMD (control group) or with EMD (25 μg/ml or 100 μg/ml). On day 3 and day 5, the cells were washed in PBS and scraped in 10 mM Tris–HCl buffer (pH = 7.6) containing 10 mM MgCl_2_ and 0.1% Triton X-100. ALP activity was determined colorimetrically with *p*-nitrophenyl phosphate as a substrate. Then, the protein content was measured by a Pierce BCA Protein Assay kit (Thermo Fisher Scientific) with the use of a microplate spectrophotometer at 405 nm and BSA as the standard. Enzyme activity was shown as Units/mg protein.

### Immunocytochemistry and confocal laser scanning microscopy

hGMSCs in eight-well chamber slides (Merck Millipore) were used for immunocytochemistry. After fixation with 4% paraformaldehyde, cells were permeabilized with methanol, blocked with 5% BSA in PBS for 20 minutes, and then exposed to the primary antibody of nestin, microtubule-associated protein 2 (MAP-2), glial fibrillary acidic protein (GFAP) (Merck Millipore), cbfα-1 (Abcam, Cambridge, UK), or OC (Epitomics Inc., Burlingame, CA, USA) overnight at 4°C. The cells were washed in PBS, exposed to the secondary antibody of FITC-conjugated goat anti-rabbit immunoglobulin G (IgG) (Abcam) or Alexa Fluor 568-conjugated goat anti-mouse IgG (InvitroGen) for 30 minutes and counterstained with 4',6-diamidino-2-phenylindole (DAPI). The nuclear translocation of cbfα-1 and protein expressions of nestin, MAP-2, GFAP and OC in hGMSCs were observed by confocal laser scanning microscopy (LSM780, Carl Zeiss MicroImaging, Inc., New York, NY, USA).

### Statistical analysis

One-way analysis of variance (ANOVA) was selected to evaluate the differences in the expression of mRNA for Cbfα-a, ALP and OC between hGMSCs treated with EMD (0, 25 or 100 μg/ml) for various times. Duncan’s test was used for *post-hoc* analysis, and *P* <0.05 was deemed to be significant.

## Results

### The stem cells could be successfully isolated from gingiva

The cell colonies could be easily identified after staining with crystal violet (Figure 1b left). At P0, the mean colony formation rate was 1.4%; however, a significantly increased colony formation rate was observed at P4 (mean = 15.9%) (Figure 1b right). Significantly higher telomerase activities, indicating a higher activity of cell renewal, were observed in hGMSCs when compared with the activity from gingival fibroblasts (Figure 1D). The isolated cells at P4 expressed high levels of surface markers for CD73, CD90 and CD105, but a low expression was observed for STRO-1 (Figure 1C). Multi-potency in osteogenic, adipogenic, and neural differentiation of the isolated hGMSCs was successfully induced (Figure 1E).

### Effect of EMD on the cell proliferation and osteogenic differentiation of hGMSCs

**Figure 2:**
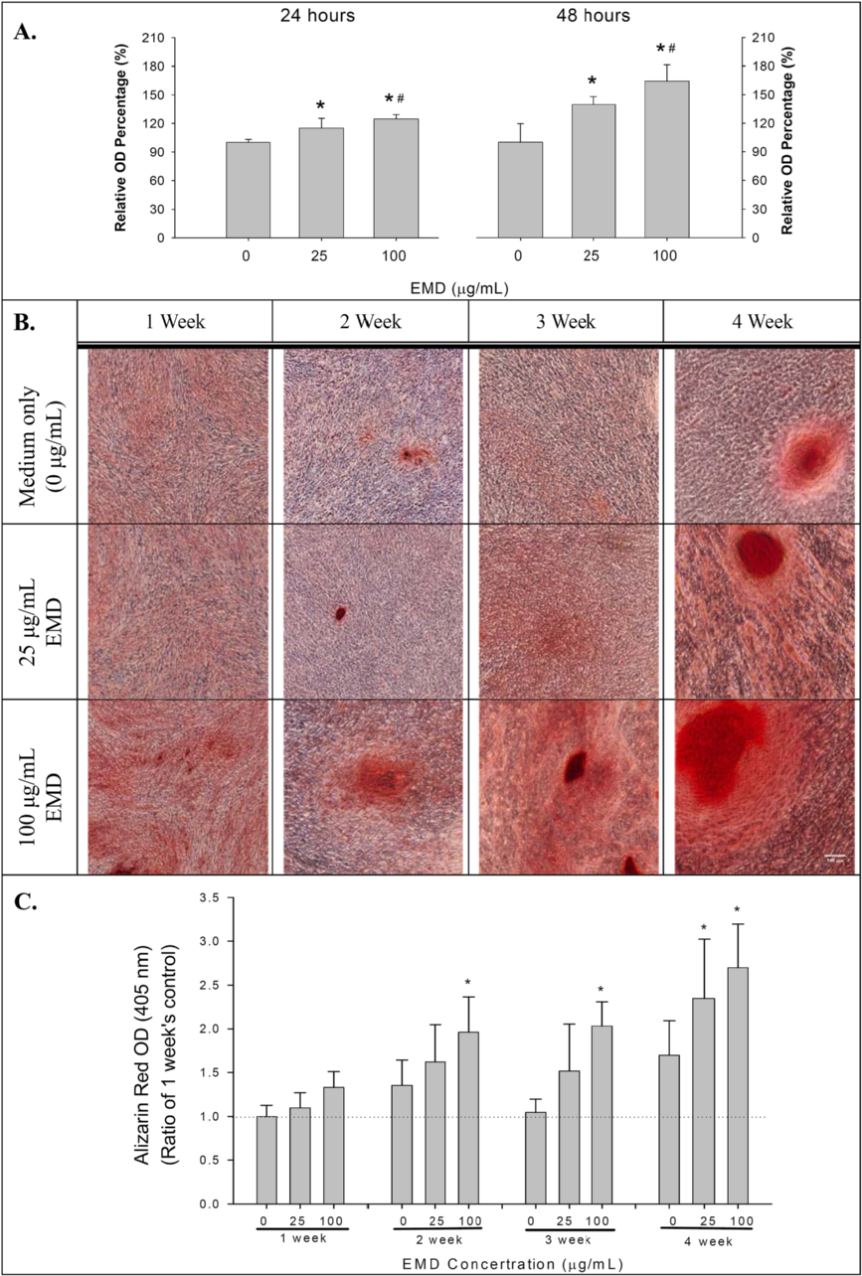
**Cellular proliferation and extracellular matrix mineralization of hGMSCs after EMD stimulation. (A)** The cellular proliferation of hGMSCs after EMD stimulation for 24 hours and 48 hours, by MTS assay (means ± standard deviations, * and #: significant difference at *P* <0.05 versus 0 and 25 μg/ml EMD, respectively). **(B)** The extracellular matrix mineralization, stained with ARS, of hGMSCs after EMD stimulation for up to four weeks. **(C)** Semi-quantitative measurments of the ARS dye from the cultured cells during the osteogenic differentiation of hGMSCs after the EMD stimulations presented in B (means ± standard deviations, and *: significant difference at *P* <0.05 versus 0 μg/ml EMD at each observation interval). ARS, Alizarin red S; EMD, enamel matrix derivative; hGMSCs, human gingiva-derived mesenchymal stem cells.

The EMD increased the number of cells in a dose dependent manner in both 24 and 48 hours (Figure 2A). The effect of EMD on the osteogenic differentiation of hGMSCs is summarized in Figure 2B-C. At the first and second week after 100 μg/mL of EMD treatment, some red nodules could be observed with ARS staining. For those wells that recieved medium only or 25 μg/mL of EMD, the nodules could only be observed three and four weeks after the treatment. The semi-quantiative measurment of the Alizarin red from the wells also showed similar findings as those shown in Figure 2B (Figure 2C).

### Effect of EMD on gene expressions of hGMSCs during osteogenic differentiation

**Figure 3:**
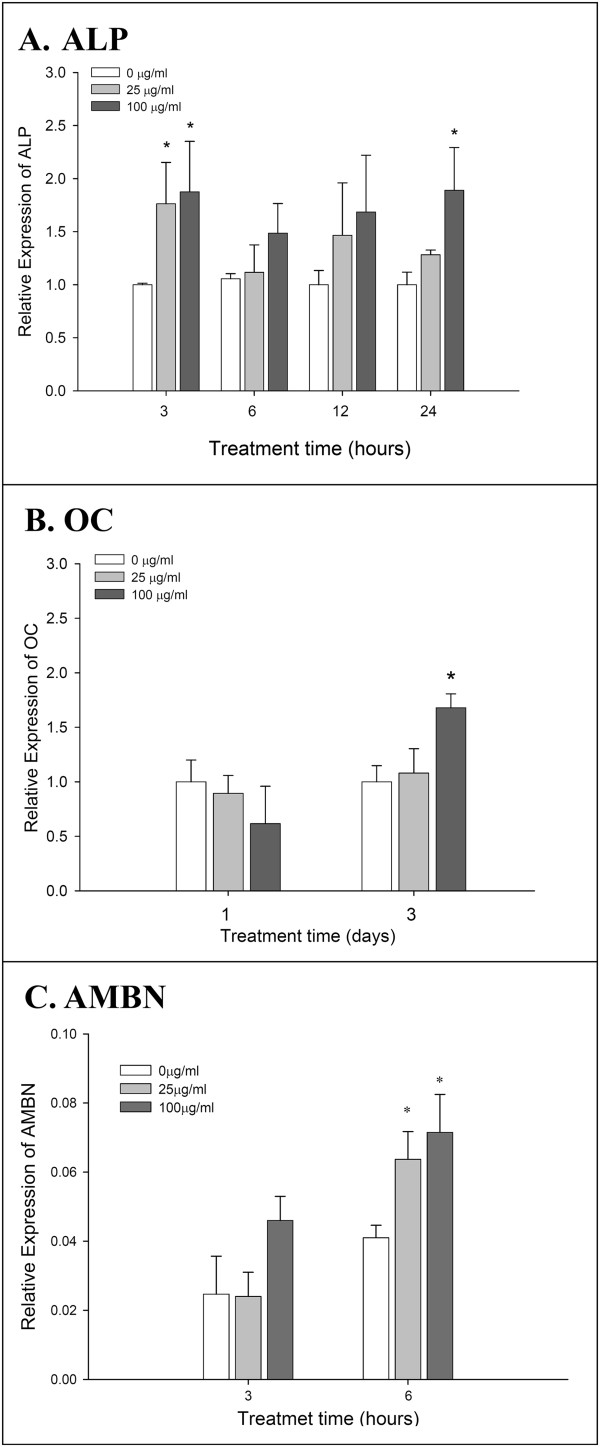
**Osteogenic gene expressions of hGMSCs after EMD stimulations. (A)** The mRNA expression of ALP in hGMSCs after EMD treatments for up to 24 hours. **(B and C)** The mRNA expressions of OC and AMBN in hGMSCs after EMD treatments. (means ± standard deviations, and *: significant difference at *P* <0.05 versus 0 μg/ml EMD at each observation interval). ALP, alkaline phosphatase; AMBN, ameloblastin; EMD, enamel matrix derivative; hGMSCs, human gingiva-derived mesenchymal stem cells; OC, osteocalcin.

Significantly increased ALP mRNA expression was observed when the cells received 25 and 100 μg/mL of EMD treatment for three hours (Figure 3A). The gene expression of OC after high dose EMD (100 μg/ml) treatments was significantly increased at day 3 (Figure 3B). Although the gene expression of AMBN was similar after EMD treatment for three hours, significantly increased expressions were observed at hour 6 (Figure 3C).

### Effect of EMD on the protein expression of hGMSCs during osteogenic differentiation

Using confocal laser scanning, increased protein expression of cbfα-1 after the EMD treatments could be observed at day 1 and nuclear translocation could be clearly observed at day 3 (Figure 3A). Increased protein expression of OC was observed at weeks 3 and 4 after the EMD treatments, especially at the 25 mg/mL concentration at week 4 (Figure 3B). Increased activities of ALP were observed at days 5 and 7 after EMD treatment (Figure 3C).

## Discussion

The discovery of stem cells and recent progress in stem cell biology has made a great contribution to the development of regenerative therapeutic strategies for multiple diseases. Generally, there are two major properties of stem cells: they are capable of both self-renewal and differentiation upon division [10]. The aim of the present study was to investigate the effects of EMD on the proliferation and mineralization of GMSCs. Our isolated human gingival cells had an increased colony forming rate, high telomerase activity, high levels of common MSC markers (for example, CD73, CD90 and CD105) and multi-potency in differentiation (Figure 1). Therefore, we suggest that the cells isolated in this study have the characteristics of MSCs. In the present study, the hGMSCs are adult mesenchymal stem cells, which are the general cell types of the tissue near where they reside. The application of adult stem cells in research and medical therapies is less controversial than embryonic stem cells because these cells can be harvested and isolated without destroying an embryo [11]. In addition, adult stem cells can be found almost throughout all body tissues, including dental tissues [6], although the proliferative potential and the differentiation capacity of adult stem cells, such as the periodontal ligament stem cells, decrease as age increases [8].

Previous *in vitro* and *in vivo* studies have shown that EMD promotes the regeneration of periodontal tissues and affects the proliferation and mineralization of cells, such as cementoblasts and periodontal ligament cells [28-34]. EMD also promotes the regulation of osteoclastogenesis, the proliferation and migration of periodontal cells, and also stimulates the signal transduction of bone morphogenic protein and transforming growth factor-β [31,35-40]. In addition, the properties of EMD are like those of the extracellular matrix protein which guide or regulate the proliferation, migration and differentiation of osteoblasts [28,30-32]. An *in vitro* study with DNA microarrays has shown that EMD is able to modulate a broad range of osteoblast biologic activity genes, such as cell cycle regulation, proliferation, apoptosis, cytoskeleton, cell adhesion, extracellular matrix production and vesicular transport in ostoeoblast cultures [28]. A recent study in a mouse preosteoblast cell line also showed that EMD increased the mRNA expression of bone sialoprotein and osteopontin, the phenotypic markers of osteoblastic differentiation; as a result, it accelerates and improves matrix mineralization [41]. In another study of human bone marrow stromal cells, EMD affected cell proliferation positively while decreasing the osteogenic differentiation [42].

**Figure 4:**
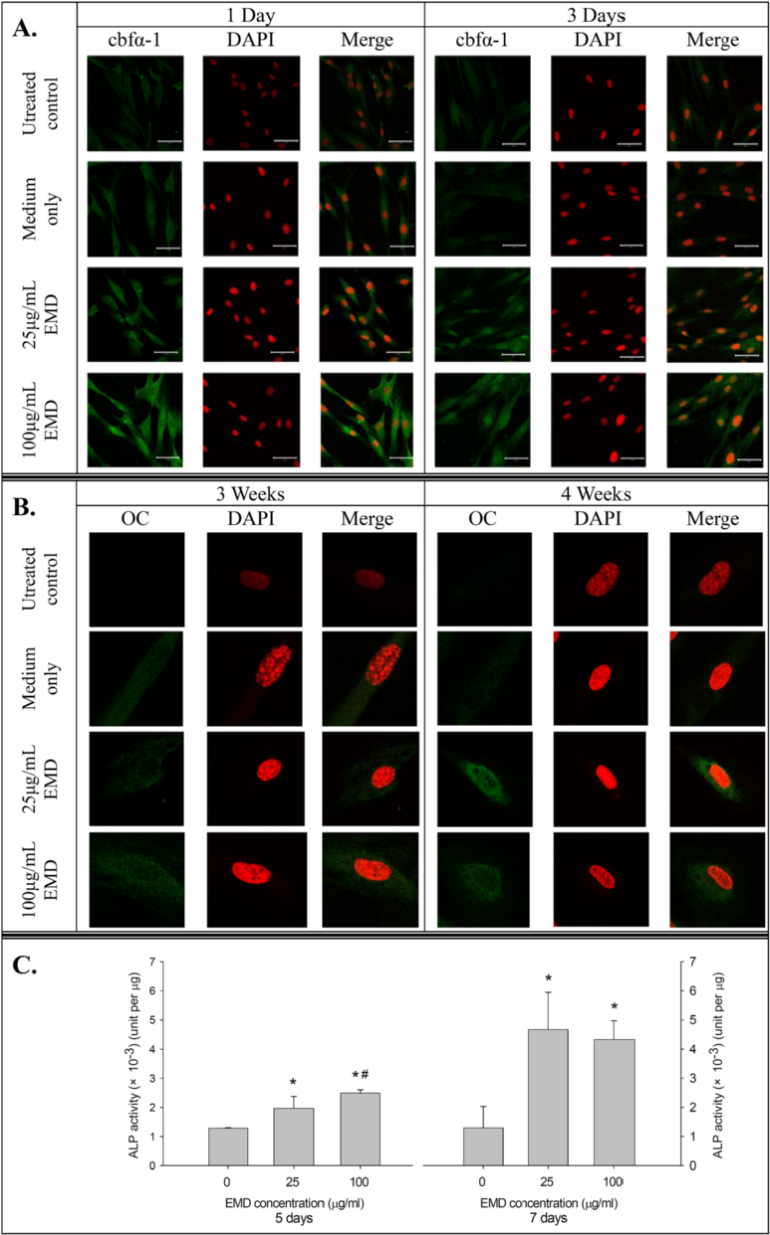
**Osteogenic protein expression of hGMSCs after EMD treatments. (A)** The protein and nuclear translocation expressions of cbfα-1 in hGMSCs after EMD treatment for three days. **(B)** The protein expression of OC in hGMSCs after EMD treatment for four weeks. **(C)** The enzyme activity of ALP in hGMSCs after EMD treatment for seven days. (means ± standard deviations, * and #: significant difference at *P* <0.05 versus 0 and 25 μg/ml EMD, respectively). ALP, alkaline phosphatase; EMD, enamel matrix derivative; hGMSCs, human gingiva-derived mesenchymal stem cells; OC, osteocalcin.

In the present study, all experiments were performed with MSCs obtained from the soft tissue of gingiva which is non-ossifying tissue. In order to demonstrate the mineralization capability of the hGMSCs, the expression of mRNA and protein for mineralization markers (for example, Cbfα-l, ALP and OC) was determined. Cbfα-l is a transcription factor, which belongs to the *runt*-domain gene family and is preferentially in the osteoblast lineage during osteogenesis. Studies have reported that Cbfα-l is an essential transcription factor for osteoblast differentiation [43]. In the present study, mRNA expression of Cbfα-l was upregulated and protein nuclear translocation was clearly observed in hGMSCs with EMD treatment (Figure 4A).ALP is a common early marker of osteogenic differentiation. Its activity was increased by EMD stimulation (Figure 4C) and, therefore, a significantly increased number of osteogenic differentiation nodules and stronger staining were observed in the extracellular matrix mineralization after being treated with EMD than without EMD (Figure 2B and C). The mineralization of extracellular matrix which is the foundation for hard tissue construction comprises two stages: preliminary synthesis of collagenous network and deposition of hydroxyapatite crystals, catalyzed by ALP. Our results demonstrate that EMD markedly assisted the mineralization of extracellular matrix.

OC is a specific late marker of osteogenic differentiation, indicating the major non-collagenic protein of the bone matrix. OC plays important regulatory functions in the bone-remodeling process, and its levels usually proceed in parallel with the final event of matrix mineralization [44,45]. In our present study, EMD significantly stimulated OC expression at all of the time points at the high dose (100 μg/ml) (Figure 3C). This finding was consistent with the increased mineralization of extracellular matrix after EMD treatment. OC was a matrix signal for bone formation, stimulating differentiation of osteoblasts.

In the present study, EMD not only enhanced the osteogenic differentiation of hGMSCs but also promoted the proliferation of hGMSCs (Figure 2a). In osteogenesis, the cell cycle progression of the osteoblast is arrested and, hence, increases its differentiation [23,46]. However, the proliferation of progenitor cells usually occurred at the early stage before the differentiation starts [47,48]. In the present study, different media were used for cell proliferation and mineralization. In the experimental hGMSC proliferation, the medium was not suitable for osteogenic differentiation and the observation period was limited to no more than 48 hours (which is at the early stage of cell proliferation). Furthermore, in the osteogenic differentiation experiment, an osteogenic medium was used and the observation period was much longer than in the proliferation experiment. As a result, hGMSCs were treated with EMD in different culture media that enhanced either proliferation or osteogenic differentiation.

## Conclusions

Our data demonstrate that hGMSCs could be successfully isolated from human gingiva, and EMD treatment could promote not only the proliferation but also the mineralization of these isolated hGMSCs.

## Abbreviations

ALP: alkaline phosphatase; AMBN: ameloblastin; BSA: bovine serum albumin; cbfα-1: core binding factor alpha; EMD: enamel matrix derivative; FBS: fetal bovine serum; GFAP: glial fibrillary acidic protein; GMSC: gingiva-derived mesenchymal stem cells; OC: osteocalcin; PBS: phosphate-uffered saline; P/S: pencillin/streptomycin.

## Competing interests

The authors declare that they have no competing interests.

## Authors' contributions

SMW, HCC, YTC, HYL, CYC, HPT and EF designed the experiments. SMW, YTC and EF wrote the manuscript. MMJF helped with the interpretation of data and contributed to the preparation/writing of the manuscript. All authors were involved in revising the manuscript. All authors have read and approved the final manuscript.
